# Supplementary material for: Rapid turnover of life-cycle-related genes in the brown algae
Source: Genome Biol. 2019 Feb 14;20:35. doi: 10.1186/s13059-019-1630-6 (PMC6374913; doi:10.1186/s13059-019-1630-6)
Supplement: Supplementary file 3 — Protein alignments, corresponding Gblocks html file and CDS sequences for the lineage conserved gametophyte-biased genes. (ZIP 2109 kb) [file 13059_2019_1630_MOESM3_ESM.zip › Con_GA_alignments/Ec-10_001830.aa_ali.fasta-gb.htm]

Ec-10\_001830.aa.fasta.trans.aa\_ali.fasta


## Gblocks 0.91b Results

Processed file: **Ec-10\_001830.aa.fasta.trans.aa\_ali.fasta**  
Number of sequences: **2**  
Alignment assumed to be: **Protein**  
New number of positions: **1139** (selected positions are underlined in blue)

```
                         10        20        30        40        50        60
                 =========+=========+=========+=========+=========+=========+
Ec-10_001830     MFITRLRRKTQIRRRKGERETLIEDAVRFQELVVSKIQERGVPVPSKLLRELQRGSEQRD
MSTRG.50322.1    MFITRLRRKTQIRRRKGGRETLAEDAVRFQDIVVSKTRARGVAVPTKLMRELQRGSEQQD
                 ############################################################


                         70        80        90       100       110       120
                 =========+=========+=========+=========+=========+=========+
Ec-10_001830     GGKRLYKLDLRRTHLTDAHVEALAQALQELPVVRKLDLRGNEAVTIQALNALNSLLKGQV
MSTRG.50322.1    GGRRLYKLDLRRIHLTDAHVEALAEALQELPVVRKLDFRGNDDVTVRTLKALNSLLKGQV
                 ############################################################


                        130       140       150       160       170       180
                 =========+=========+=========+=========+=========+=========+
Ec-10_001830     ELFRLVQADASLADSSLSFLYSVKLDATVLSLDRRATEELEAQTAALEVADSVLDVRHAF
MSTRG.50322.1    ELFRLVQADASLADSSLSFLYSVKLDPVVLSLDKAASEELEAHTAALEVADSVLDVRHAF
                 ############################################################


                        190       200       210       220       230       240
                 =========+=========+=========+=========+=========+=========+
Ec-10_001830     FQADVRGSGTLTEHELSGAMRIVMGVTPKAAETSRVLRFFGAPGAQGNDPNVINLGGFEA
MSTRG.50322.1    FEADIRESGTLTEHELSDAMRIVMGSTPKAGETSRVLRFFGEPGPEGKDPTMINLGGFEA
                 ############################################################


                        250       260       270       280       290       300
                 =========+=========+=========+=========+=========+=========+
Ec-10_001830     AMVGRLRESHTLPVLPQRRDDLLFGSAGDKEGQ-ESLECFSPWSTRSFGATTGGIR----
MSTRG.50322.1    AMVGRLRDSHTLPMLPQGCDELLFGSVEDRGDQKENPECFSPWSTRSFGATVGSILDGKG
                 ################################# #####################     


                        310       320       330       340       350       360
                 =========+=========+=========+=========+=========+=========+
Ec-10_001830     GDKVGGGGVGNKRSPLSASQKLSPGAGMGTHVLSLPPWRGGEGGSELSNTQNDDEGQSQR
MSTRG.50322.1    SGKGGGGGIGHKRSPLSASQDLRSAAGMRTQVPPLPPWRGGACGSELERTRKDNEGEGGR
                   ##########################################################


                        370       380       390       400       410       420
                 =========+=========+=========+=========+=========+=========+
Ec-10_001830     AQRGDADLENIFDDDNCDEVDKNDGAEDAAAPRRVRETSRTSTRNSGNGRERSNRGARSS
MSTRG.50322.1    GAENNVELEDIFNDDGGNGSGEQD-VSRRSAPKAGPAIVRSGDDRSGREKGEYSRRSSTS
                 ########################      ##############################


                        430       440       450       460       470       480
                 =========+=========+=========+=========+=========+=========+
Ec-10_001830     TSSLATDAREEKEERHQEPARQRRQKEGVALVAHENSELMSSAARLAGSRFLNSDGGGDT
MSTRG.50322.1    TSIAAETHEDGAGRRSSGATEEKRPKKEINSLQLKD--GFEDTPRL-------SGYGGHG
                 #####         ############                           #######


                        490       500       510       520       530       540
                 =========+=========+=========+=========+=========+=========+
Ec-10_001830     EDDVLSDCSSDDDLSCTSARAQSTGKGTVNQNGGR-LRTEHPNFSREETGGGGGGGDPKS
MSTRG.50322.1    EESVLTEGSSDEESPRDSPWAQSR-KEMVTEDGSRPWSDEHPNHPSDEIGTAGLSDWRRH
                 #######################  ##########    ##############       


                        550       560       570       580       590       600
                 =========+=========+=========+=========+=========+=========+
Ec-10_001830     NTEKTSNWR-RHSSNPAAQGGPRFDDRSASGSSSGGGGRSVCFSEDRASGDRRVSDASRS
MSTRG.50322.1    SSGPTPTVQGRQGSNNSTVSGSRNS-RSSSSSGNGSDGRKVSLPKGESSAGRRVSDTPRI
                           #############   ##################################


                        610       620       630       640       650       660
                 =========+=========+=========+=========+=========+=========+
Ec-10_001830     SHSGDDSALEPAGSGASTHTNPGRRHSILKGNQGRDDGVRRNFGDETPPSTPQTASVPGR
MSTRG.50322.1    CRLEVQSAQAKAKTGATTSKISERRPSDLKGDHDNGDAFTGSAGNKTGPLTKGQSPQTRR
                 ############################################################


                        670       680       690       700       710       720
                 =========+=========+=========+=========+=========+=========+
Ec-10_001830     GDAVVTEPATASRSNAVRAHQTGGVRTNLI-SEEGNVTVIGMTEPHKEAATSSAASSRAE
MSTRG.50322.1    DGAAVDYKSPHSSVSAS--CQAGTDETDVLRREEKDAAALGSIKGIAGGFICSPPLSAAE
                 ################                                    ########


                        730       740       750       760       770       780
                 =========+=========+=========+=========+=========+=========+
Ec-10_001830     PPGEEPATRSRLAQASPLTTTNGGGSGMPAGRVLLKASDRDPHPRGVVIVDGETG-----
MSTRG.50322.1    AVGAGAAKQRGNSSMSPPTTPDGGTVSIPAGQATPESSRGETNPTRGVSLSGDDGGGQSE
                 #######################################################     


                        790       800       810       820       830       840
                 =========+=========+=========+=========+=========+=========+
Ec-10_001830     --TSGVPMKAEAASNGYREERDATLG----EEATPSLVETSETVTIGARPAALSSMSAVP
MSTRG.50322.1    MVESGTFHNVAASAAGATAGKEGRGGKYTTAETHTACLREGLGVAMARSPSVISPASVAV
                    #############                           #################


                        850       860       870       880       890       900
                 =========+=========+=========+=========+=========+=========+
Ec-10_001830     ATAATGVVTSTEAVTAKKQEQHEEEVGP----TTATLCEQRRRAEDAGDPLVVPTP-SLE
MSTRG.50322.1    AAVARGEPTRKDAGAVARKKEPKQEHEPARIIVRESLSEQRQPVEERDSSSLTSTPRSSD
                 #############                                               


                        910       920       930       940       950       960
                 =========+=========+=========+=========+=========+=========+
Ec-10_001830     FSG-----GNRLVAMSAETAEPNTSLGESRCGGTSPGAAANSN------------AAAES
MSTRG.50322.1    QRDGEHNPSVTNIAASTNPAQSDTSPKRSKSGEATVSAAAAVAGVVVPAQDVWPPRNAQV
                              ###########################                    


                        970       980       990      1000      1010      1020
                 =========+=========+=========+=========+=========+=========+
Ec-10_001830     QVRGG-VVRDSVSIADPKRVSAPQGVATTLLHASSVNSTGARTPGGAGGVLENDRVGRPA
MSTRG.50322.1    NARGAGPATNDLATSEPDTLRSHSGRTGALPQNVRVHGTGVLVGGGDGDFAE-ERRNAPV
                                 ####################################        


                       1030      1040      1050      1060      1070      1080
                 =========+=========+=========+=========+=========+=========+
Ec-10_001830     GL--------SLEAIVEAPGSGGGGDG--------GDAPAVAAAGASTLALDYSG-----
MSTRG.50322.1    RMSGDAMEASPLEAISEGPGEASSGDGSASGDARGGTAQTVATADAFPLDLDCGGGVGVG
                            ################        ####################     


                       1090      1100      1110      1120      1130      1140
                 =========+=========+=========+=========+=========+=========+
Ec-10_001830     -SGGGNSGPIRMPYCLSPTNVVRQARGGSGAAATAGSSCRERESLAARIQRLGDINAAAT
MSTRG.50322.1    ASGVCGVGAMRKPYCLSPTNVTRQFQAGSDARAATGAGCRERERLAARIQLSADNEVAAV
                  ###########################################################


                       1150      1160      1170      1180      1190      1200
                 =========+=========+=========+=========+=========+=========+
Ec-10_001830     ANAVNPAAQNCSVSESGFAALSGHSEGGGGAAGGSPQ--FFANAEHPRLPSSGGMPTPPS
MSTRG.50322.1    AAAAPFVNPASQGNPSGLSPSTSPGKASGADCGFALEDRDTSSSIGNRLAQANDVSARPL
                 ###                                                         


                       1210      1220      1230      1240      1250      1260
                 =========+=========+=========+=========+=========+=========+
Ec-10_001830     SS-----HGGMSPER-SRSCGLLIQD---G--GGGGSYCSSPSPSSRFHLPSSETKTAPL
MSTRG.50322.1    SVGRNPRAGAGSPDHGDRSSGRWHVDGGDGPGTGGGSSFSSPSPPKRFYLPSSETKTAPL
                                                  ###########################


                       1270      1280      1290      1300      1310      1320
                 =========+=========+=========+=========+=========+=========+
Ec-10_001830     YSAGGSTSLFEGQVLRADSEPVLRR--------------AAEMGSET--KGKAGGGEGRE
MSTRG.50322.1    CGVGGATSLFEGSLATLNKSTASFRGGREEAWCFSRVDFEAGFRGDTGVETKGGGGEVKD
                 ############                                       #########


                       1330      1340      1350      1360      1370      1380
                 =========+=========+=========+=========+=========+=========+
Ec-10_001830     TDGMLSSPAGALQLDEEQLGLRINEGALVITRTGLSKMSTVLQLEAVYGYGRESLIGLHT
MSTRG.50322.1    GAGLESSLEGVLREDEEQLAIRLNDGGLVISRSGLAKMGVILELETVQERGRDCLIGLHT
                 ############################################################


                       1390      1400      1410      1420      1430      1440
                 =========+=========+=========+=========+=========+=========+
Ec-10_001830     LVLSYNQLQVLDISVLTTMPALRHLDVSHNLLCRMEPMDGRDLPPRLETLNMSHNRISRI
MSTRG.50322.1    LVLSCNRLQVLDISVLAAMPSLRHLDVSHNLLCRMEPMEGRDLPPRLETLNMSHNRISRI
                 ############################################################


                       1450      1460      1470      1480      1490      1500
                 =========+=========+=========+=========+=========+=========+
Ec-10_001830     GGIAQCFLLRALDLRHNRIKRVQGLEHLSLLLQLDLGHNFISKAASARALSFNRSLKLLW
MSTRG.50322.1    GGIGQCFLLRVVDLRNNRIKRVQGLEHLSLLEQLDLRHNLISKAASARALSFNRSLKLLW
                 ############################################################


                       1510      1520      1530      1540      1550      1560
                 =========+=========+=========+=========+=========+=========+
Ec-10_001830     LEGNPLARHPRYRPTLTCLLPHVRSIDSRAMPPSSDSDQRRWEAGGGSADEEEGGDGCSS
MSTRG.50322.1    LTGNPLARHPRYRPTLTCLLPHVRSIDSRGMPPSSDSEQRRWTAE---------------
                 ############################################                


                       1570      1580      1590      1600      1610      1620
                 =========+=========+=========+=========+=========+=========+
Ec-10_001830     TVAAAGHAAAARGHQRGRSGNTSASREWQAEQDTRRSKEWQQVMEWRKGQARREEEAKGQ
MSTRG.50322.1    ------------------------------------------------------------
                                                                             


                       1630      1640      1650      1660      1670      1680
                 =========+=========+=========+=========+=========+=========+
Ec-10_001830     ALEGRHDVRQAINAAQQRRQAEELARPRPRKCPSEEAISKAKEVACRRPAVVFGISYRGE
MSTRG.50322.1    ------------------------------------------------------------
                                                                             


                       1690      1700      1710      1720      1730      1740
                 =========+=========+=========+=========+=========+=========+
Ec-10_001830     RERGTHASDAAYAASAGMDTANTPPSPPRSKGHQQHGRLFDVAADGYELRGKGVGGGCGG
MSTRG.50322.1    ------------------------------------------------------------
                                                                             


                       1750      1760      1770      1780      1790      1800
                 =========+=========+=========+=========+=========+=========+
Ec-10_001830     GMDRREEYIQSPHGAARENGAVSVRDVLGRQEWTTTDPPLQDNDESLQQAYQNSGSDAPP
MSTRG.50322.1    ------------------------------------------------------------
                                                                             


                       1810      1820      1830      1840      1850      1860
                 =========+=========+=========+=========+=========+=========+
Ec-10_001830     PSYLLPSSRGRSSKRSSVGAVEGHDSPTPSVPVMMTVDSDGAATARTTVRAKKAPEDIEG
MSTRG.50322.1    ------------------------------------------------------------
                                                                             


                       1870      1880      1890      1900      1910      1920
                 =========+=========+=========+=========+=========+=========+
Ec-10_001830     ASAESSGGAGHERNELVDSWLLDVKLETETAGTALQVLLRMCKERGGDTDGERRRLSSFR
MSTRG.50322.1    ------------------------------------------------------------
                                                                             


                       1930      1940      1950      1960      1970      1980
                 =========+=========+=========+=========+=========+=========+
Ec-10_001830     TALEGMGLFSPQHGPTPTEFVGDEAGVLAHKVAAAARQVAMTKAAVKNLLRLMESVEPGE
MSTRG.50322.1    ------------------------------------------------------------
                                                                             


                       1990      2000      2010      2020      2030      2040
                 =========+=========+=========+=========+=========+=========+
Ec-10_001830     DGKAELDQYAKFIRLQREIMIGVGGHPNADTSSPETKREPTTSAAVVQQGENANCHRSLP
MSTRG.50322.1    ------------------------------------------------------------
                                                                             


                       2050      2060      2070      2080      2090      2100
                 =========+=========+=========+=========+=========+=========+
Ec-10_001830     EQGEPEGRASGNPGGGVALPSSSSLYATATTAAREGPTSQDLNDVSGSEERVMTTGTEGI
MSTRG.50322.1    ------------------------------------------------------------
                                                                             


                       2110      2120      2130      2140      2150      2160
                 =========+=========+=========+=========+=========+=========+
Ec-10_001830     VVPPCGGAESVHETDANDVIVPLGLGPEASARCTGGAIDAASKDVPGTFQSITCEPLRPA
MSTRG.50322.1    ------------------------------------------------------------
                                                                             


                       2170      2180      2190      2200      2210      2220
                 =========+=========+=========+=========+=========+=========+
Ec-10_001830     EEVVTEEALVTMVVGITQGKDSSSLLPSNQDSVVVAGIVDQSSISAGVSEIASAVGFVHP
MSTRG.50322.1    ------------------------------------------------------------
                                                                             


                       2230      2240      2250      2260      2270      2280
                 =========+=========+=========+=========+=========+=========+
Ec-10_001830     KCTSANKEKNEGREASQDLGDYAGGNPLISTSPAVVSADETSTSTPRVTIDGTPSDSSLK
MSTRG.50322.1    ------------------------------------------------------------
                                                                             


                       2290      2300      2310      2320      2330      2340
                 =========+=========+=========+=========+=========+=========+
Ec-10_001830     PNGTSVLRTDNGDDADATAEVAEVAEVPGKRKEEGGEGLSATPVTSATSTANLSARERLL
MSTRG.50322.1    ------------------------------------------------------------
                                                                             


                       2350      2360      2370      2380      2390      2400
                 =========+=========+=========+=========+=========+=========+
Ec-10_001830     RHVRATASPRSGSDDDSSCCSSGGGSGTDNNDSESKTVRRLPTTEGLQRERDIILAQEDG
MSTRG.50322.1    ----------------------------------------------------VLAGEEDG
                                                                             


                       2410      2420      2430      2440      2450      2460
                 =========+=========+=========+=========+=========+=========+
Ec-10_001830     GEEGCDGEVAAGGDGGEDIEIVGVAKQEEGVENSRTREEGGSVDGRPMTTRPATRWEGEG
MSTRG.50322.1    GDW---------------------------------------------------------
                                                                             


                       2470      2480      2490      2500      2510      2520
                 =========+=========+=========+=========+=========+=========+
Ec-10_001830     GTEGNDKNELGVSSNASRQPDVPSASPATQLANPLATAEAGSIVIFDKDGSVSSGTPAGI
MSTRG.50322.1    ------------------------------------------------------------
                                                                             


                       2530      2540      2550      2560      2570      2580
                 =========+=========+=========+=========+=========+=========+
Ec-10_001830     SGDVYRVEVDVSAIDTPTAAGAGFSPTSEHQTSHIEAESDTGTVPSTDAVGSGVDCTAVS
MSTRG.50322.1    ------------------------------------------------------------
                                                                             


                       2590      2600      2610      2620      2630      2640
                 =========+=========+=========+=========+=========+=========+
Ec-10_001830     SAATPAVVCPSTIASDAIIDENGGELRGSTLDESGEVAQGTTKHDNDSEQILSHPVSDVS
MSTRG.50322.1    ------------------------------------------------------------
                                                                             


                       2650      2660      2670      2680      2690      2700
                 =========+=========+=========+=========+=========+=========+
Ec-10_001830     TPIRSDLHVAGNEKDDKAKGSVEVATAGNESTLGNSSEKAKPDSGRSDEAATTTCGEAEE
MSTRG.50322.1    ------------------------------------------------------------
                                                                             


                       2710      2720      2730      2740      2750      2760
                 =========+=========+=========+=========+=========+=========+
Ec-10_001830     NAEEEVNTGGDANTKTSNERDVKLDGAENETAPADPTTELAWIEGYDPGHDCYYYHHVPT
MSTRG.50322.1    ------------------------------------------------------------
                                                                             


                       2770      2780      2790      2800      2810      2820
                 =========+=========+=========+=========+=========+=========+
Ec-10_001830     GESRWYKPDEPYESYVHSDEEDGDGGTSYSLRDEGGTPITATTAAALEDDNLRPERKERR
MSTRG.50322.1    ------------------------------------------------------------
                                                                             


                       2830      2840      2850      2860      2870      2880
                 =========+=========+=========+=========+=========+=========+
Ec-10_001830     KDDENVDNQHLRGKRGGIEEEEATEATTRRSSLSRKVKEPSSGKRRSSKSSTASAGSQGR
MSTRG.50322.1    ------------------------------------------------------------
                                                                             


                       2890      2900      2910      2920      2930      2940
                 =========+=========+=========+=========+=========+=========+
Ec-10_001830     RRDDDDGGARDHSRSGRASRQQRGKTALERLNDFTDDNTCGSDDPRSDGYGCERRRSSGS
MSTRG.50322.1    ------------------------------------------------------------
                                                                             


                       2950      2960      2970      2980      2990      3000
                 =========+=========+=========+=========+=========+=========+
Ec-10_001830     SRRQDGTGSRRHRDDDDDRYRSRRKHSSGDGGSRHRKSRGDSSLRTEGYRDDGKRDHDRR
MSTRG.50322.1    ------------------------------------------------------------
                                                                             


                       3010      3020      3030      3040      3050
                 =========+=========+=========+=========+=========+=
Ec-10_001830     RPYSQRRSSDSRYILSSDGSRNSDDDEGALMGRTSSARDRGGSRSRRSSAR
MSTRG.50322.1    ---------------------------------------------------
```

```
Parameters used
Minimum Number Of Sequences For A Conserved Position: 2
Minimum Number Of Sequences For A Flanking Position: 2
Maximum Number Of Contiguous Nonconserved Positions: 8
Minimum Length Of A Block: 10
Allowed Gap Positions: None
Use Similarity Matrices: Yes
```

```
Flank positions of the 20 selected block(s)
Flanks: [1  273]  [275  295]  [303  384]  [391  425]  [435  446]  [474  503]  [506  515]  [520  533]  [551  563]  [567  676]  [713  775]  [784  796]  [824  853]  [914  940]  [977  1012]  [1032  1047]  [1056  1075]  [1082  1143]  [1234  1272]  [1312  1544]  

New number of positions in Ec-10_001830.aa.fasta.trans.aa_ali.fasta-gb:  1139  (37% of the original 3051 positions)
```
